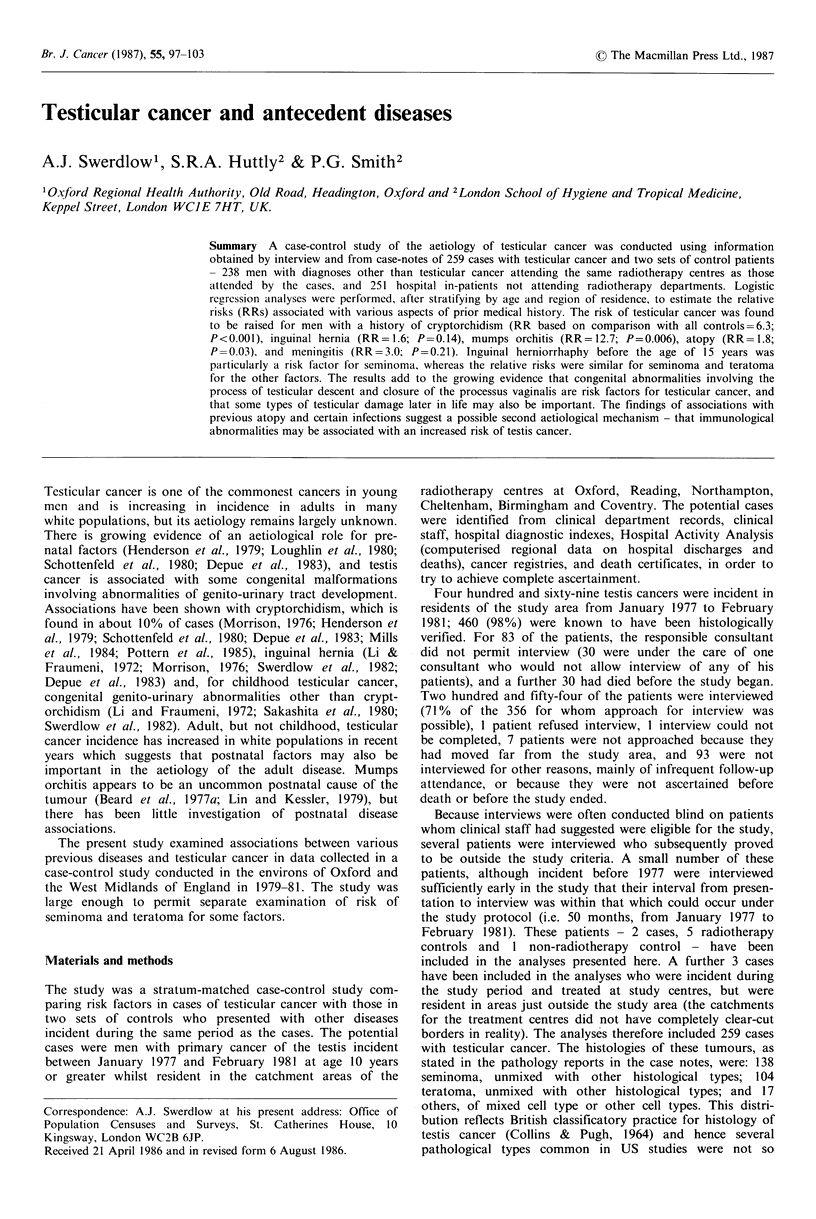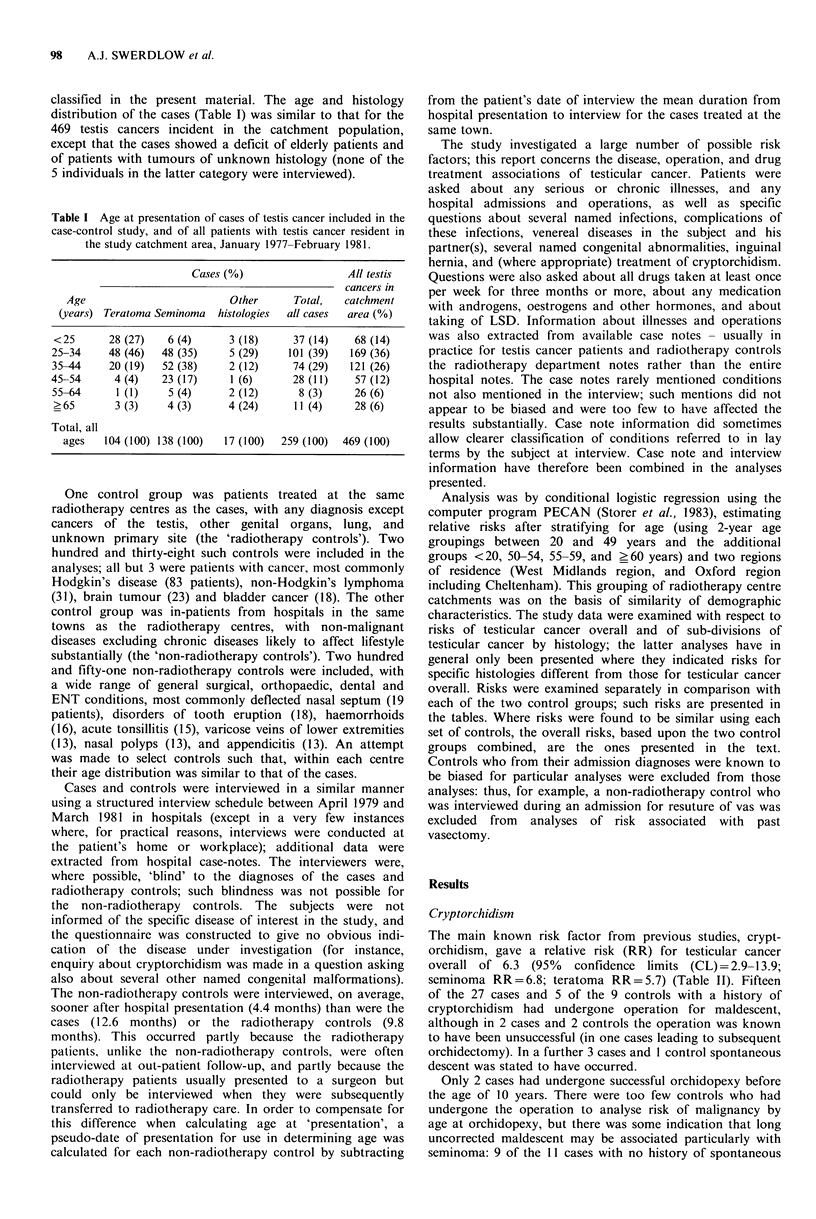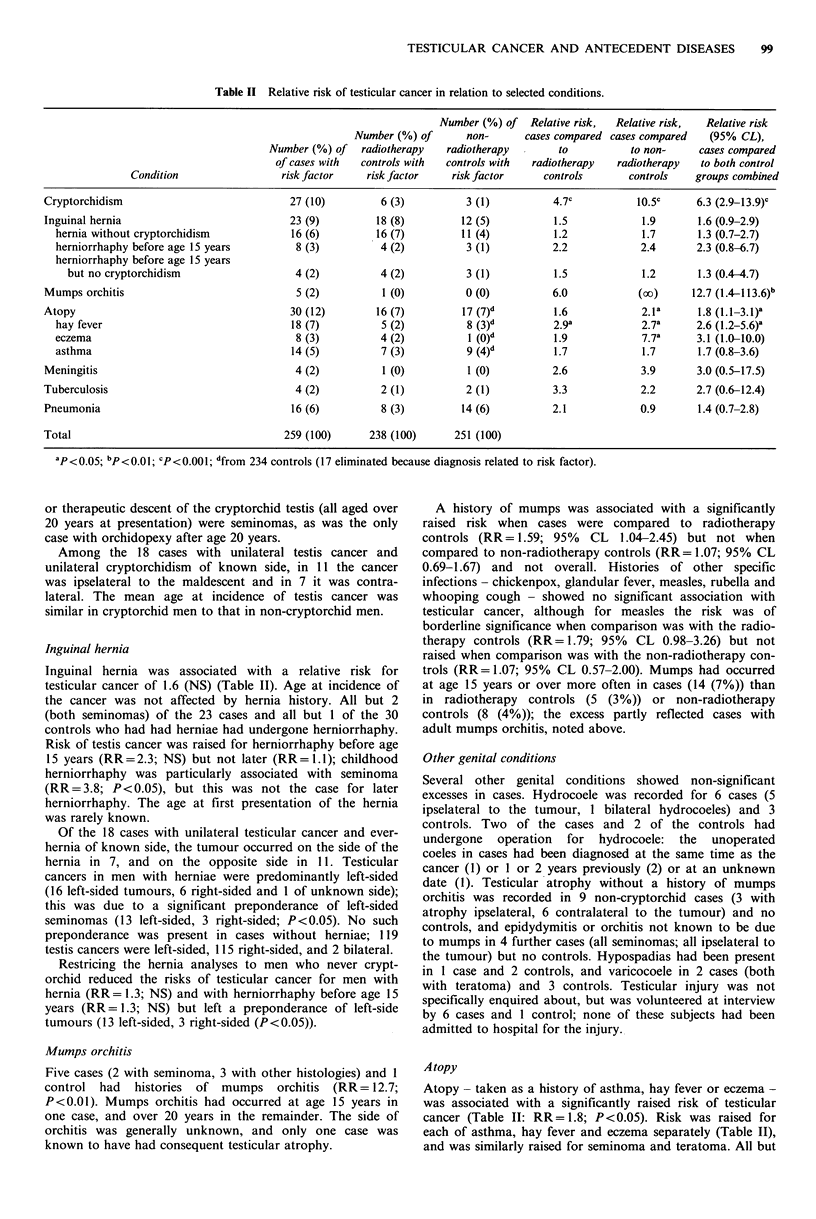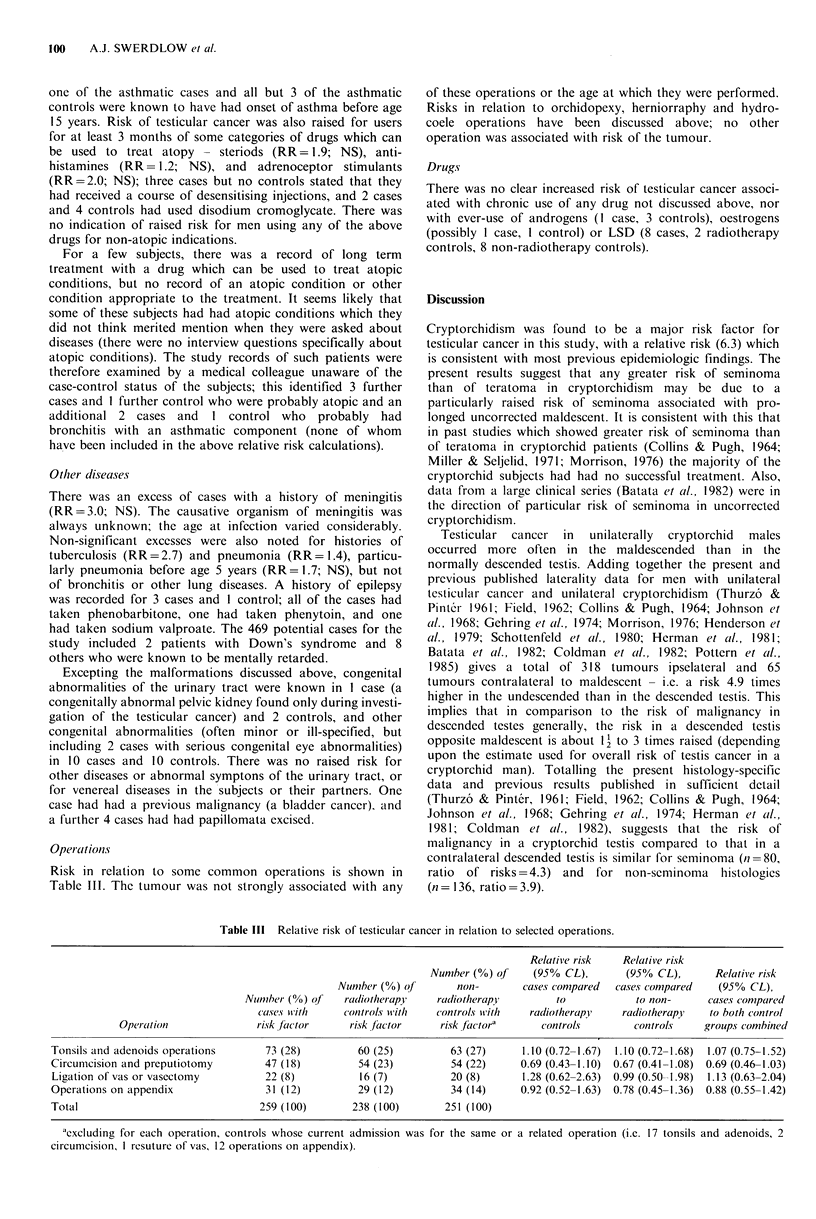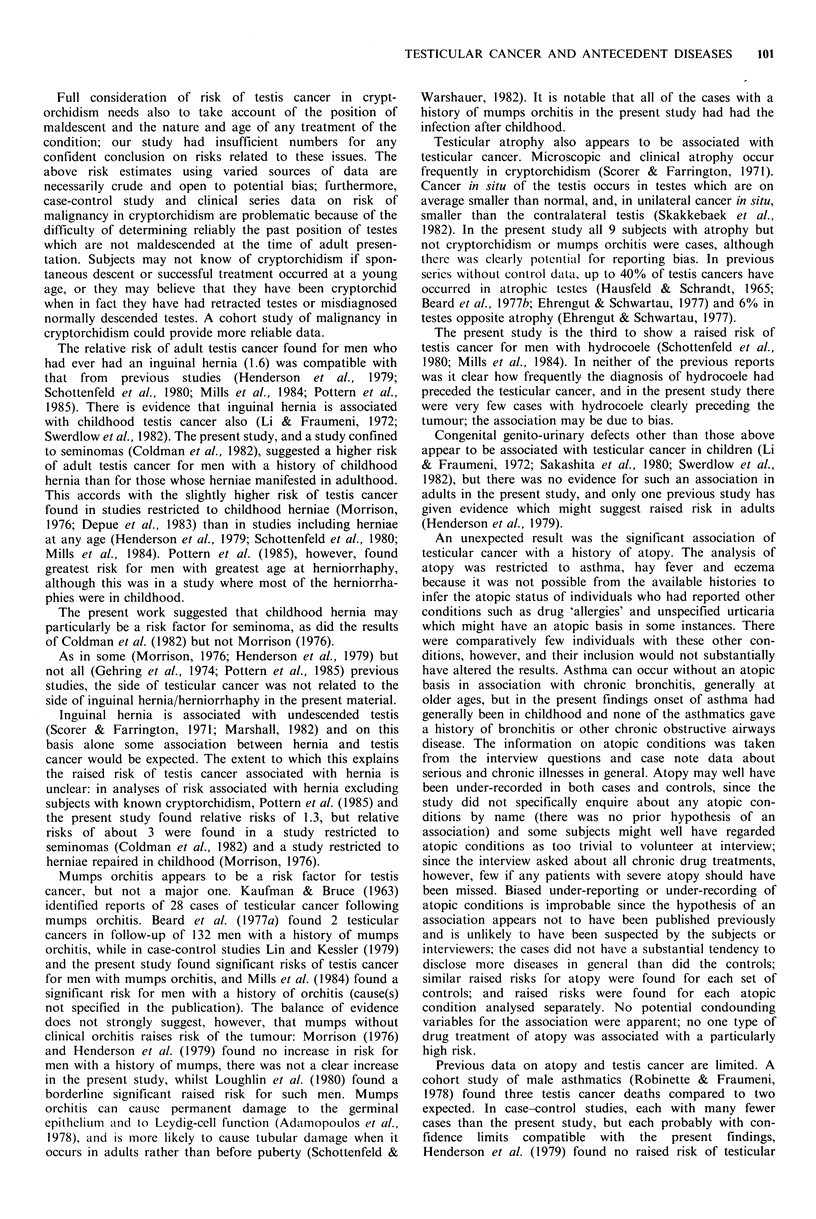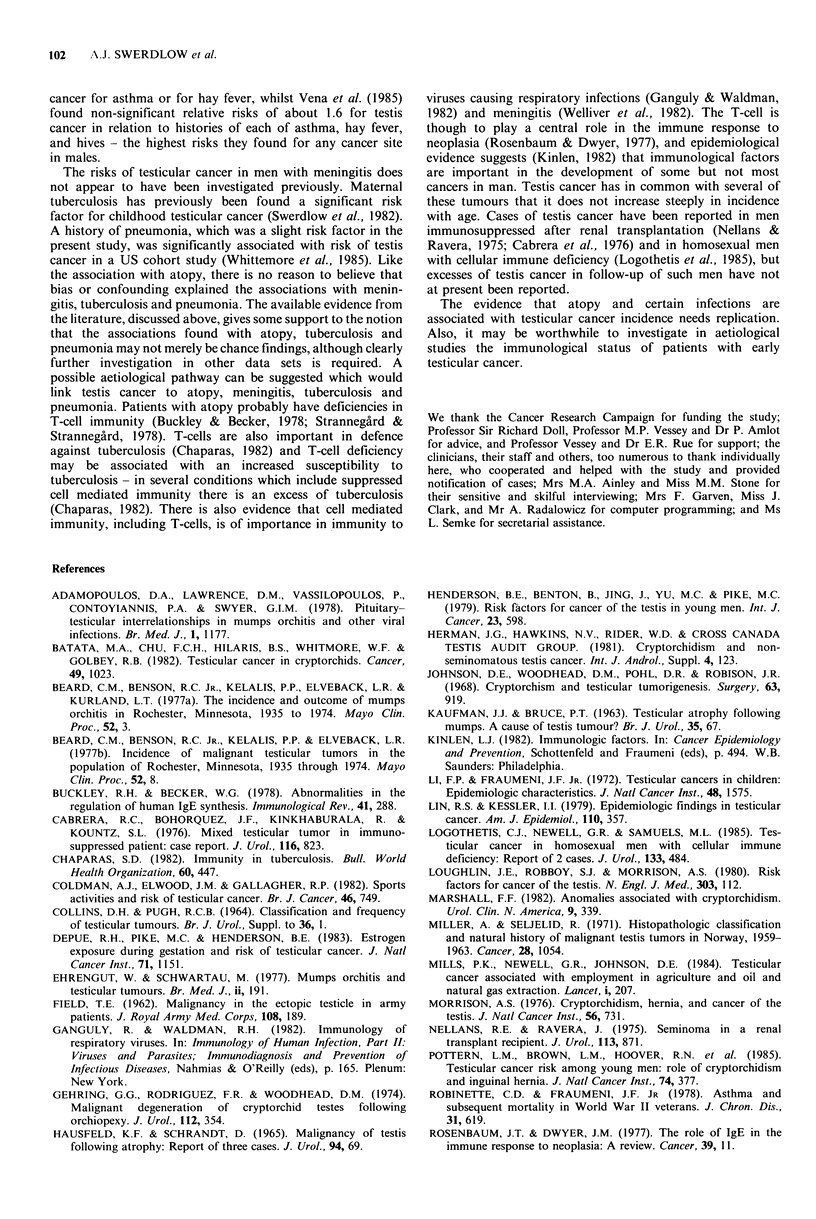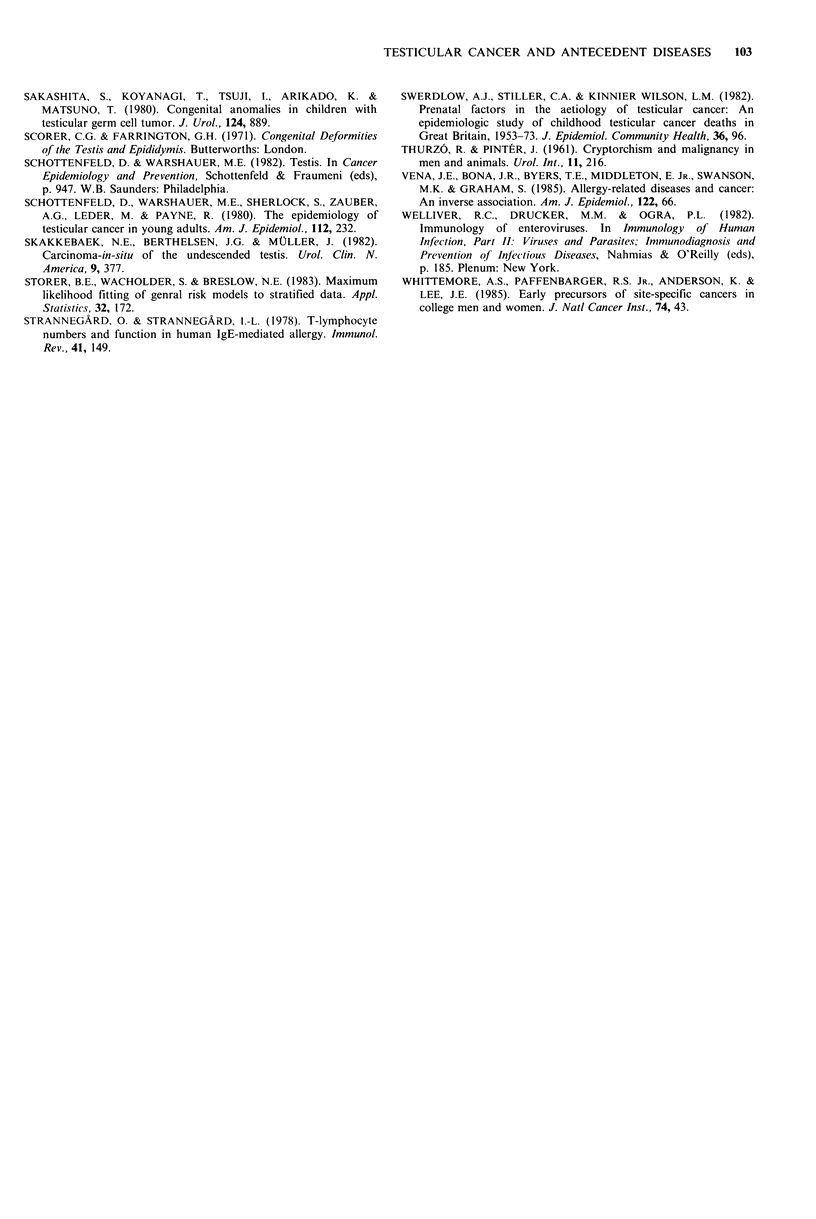# Testicular cancer and antecedent diseases.

**DOI:** 10.1038/bjc.1987.20

**Published:** 1987-01

**Authors:** A. J. Swerdlow, S. R. Huttly, P. G. Smith

## Abstract

A case-control study of the aetiology of testicular cancer was conducted using information obtained by interview and from case-notes of 259 cases with testicular cancer and two sets of control patients -238 men with diagnoses other than testicular cancer attending the same radiotherapy centres as those attended by the cases, and 251 hospital in-patients not attending radiotherapy departments. Logistic regression analyses were performed, after stratifying by age and region of residence, to estimate the relative risks (RRs) associated with various aspects of prior medical history. The risk of testicular cancer was found to be raised for men with a history of cryptorchidism (RR based on comparison with all controls = 6.3; P less than 0.001), inguinal hernia (RR = 1.6; P = 0.14), mumps orchitis (RR = 12.7; P = 0.006), atopy (RR = 1.8; P = 0.03), and meningitis (RR = 3.0; P = 0.21). Inguinal herniorrhaphy before the age of 15 years was particularly a risk factor for seminoma, whereas the relative risks were similar for seminoma and teratoma for the other factors. The results add to the growing evidence that congenital abnormalities involving the process of testicular descent and closure of the processus vaginalis are risk factors for testicular cancer, and that some types of testicular damage later in life may also be important. The findings of associations with previous atopy and certain infections suggest a possible second aetiological mechanism - that immunological abnormalities may be associated with an increased risk of testis cancer.


*Br. J. Cancer* (1987), **55**, 97–103 © The Macmillan Press Ltd., 1987

### Testicular cancer and antecedent diseases

A.J. Swerdlow[1], S.R.A. Huttly[2] & P.G. Smith[2]

[1]*Oxford Regional Health Authority, Old Road, Headington, Oxford and* [2]*London School of Hygiene and Tropical Medicine, Keppel Street, London WC1E 7HT, UK.*

**Summary** A case-control study of the aetiology of testicular cancer was conducted using information obtained by interview and from case-notes of 259 cases with testicular cancer and two sets of control patients – 238 men with diagnoses other than testicular cancer attending the same radiotherapy centres as those attended by the cases, and 251 hospital in-patients not attending radiotherapy departments. Logistic regression analyses were performed, after stratifying by age and region of residence, to estimate the relative risks (RRs) associated with various aspects of prior medical history. The risk of testicular cancer was found to be raised for men with a history of cryptorchidism (RR based on comparison with all controls = 6.3; $P<0.001$), inguinal hernia (RR = 1.6; $P=0.14$), mumps orchitis (RR = 12.7; $P=0.006$), atopy (RR = 1.8; $P=0.03$), and meningitis (RR = 3.0; $P=0.21$). Inguinal herniorrhaphy before the age of 15 years was particularly a risk factor for seminoma, whereas the relative risks were similar for seminoma and teratoma for the other factors. The results add to the growing evidence that congenital abnormalities involving the process of testicular descent and closure of the processus vaginalis are risk factors for testicular cancer, and that some types of testicular damage later in life may also be important. The findings of associations with previous atopy and certain infections suggest a possible second aetiological mechanism – that immunological abnormalities may be associated with an increased risk of testis cancer.

Testicular cancer is one of the commonest cancers in young men and is increasing in incidence in adults in many white populations, but its aetiology remains largely unknown. There is growing evidence of an aetiological role for prenatal factors (Henderson *et al.*, 1979; Loughlin *et al.*, 1980; Schottenfeld *et al.*, 1980; Depue *et al.*, 1983), and testis cancer is associated with some congenital malformations involving abnormalities of genito-urinary tract development. Associations have been shown with cryptorchidism, which is found in about 10% of cases (Morrison, 1976; Henderson *et al.*, 1979; Schottenfeld *et al.*, 1980; Depue *et al.*, 1983; Mills *et al.*, 1984; Pottern *et al.*, 1985), inguinal hernia (Li & Fraumeni, 1972; Morrison, 1976; Swerdlow *et al.*, 1982; Depue *et al.*, 1983) and, for childhood testicular cancer, congenital genito-urinary abnormalities other than cryptorchidism (Li and Fraumeni, 1972; Sakashita *et al.*, 1980; Swerdlow *et al.*, 1982). Adult, but not childhood, testicular cancer incidence has increased in white populations in recent years which suggests that postnatal factors may also be important in the aetiology of the adult disease. Mumps orchitis appears to be an uncommon postnatal cause of the tumour (Beard *et al.*, 1977*a*; Lin and Kessler, 1979), but there has been little investigation of postnatal disease associations.

The present study examined associations between various previous diseases and testicular cancer in data collected in a case-control study conducted in the environs of Oxford and the West Midlands of England in 1979–81. The study was large enough to permit separate examination of risk of seminoma and teratoma for some factors.

### Materials and methods

The study was a stratum-matched case-control study comparing risk factors in cases of testicular cancer with those in two sets of controls who presented with other diseases incident during the same period as the cases. The potential cases were men with primary cancer of the testis incident between January 1977 and February 1981 at age 10 years or greater whilst resident in the catchment areas of the radiotherapy centres at Oxford, Reading, Northampton, Cheltenham, Birmingham and Coventry. The potential cases were identified from clinical department records, clinical staff, hospital diagnostic indexes, Hospital Activity Analysis (computerised regional data on hospital discharges and deaths), cancer registries, and death certificates, in order to try to achieve complete ascertainment.

Four hundred and sixty-nine testis cancers were incident in residents of the study area from January 1977 to February 1981; 460 (98%) were known to have been histologically verified. For 83 of the patients, the responsible consultant did not permit interview (30 were under the care of one consultant who would not allow interview of any of his patients), and a further 30 had died before the study began. Two hundred and fifty-four of the patients were interviewed (71% of the 356 for whom approach for interview was possible), 1 patient refused interview, 1 interview could not be completed, 7 patients were not approached because they had moved far from the study area, and 93 were not interviewed for other reasons, mainly of infrequent follow-up attendance, or because they were not ascertained before death or before the study ended.

Because interviews were often conducted blind on patients whom clinical staff had suggested were eligible for the study, several patients were interviewed who subsequently proved to be outside the study criteria. A small number of these patients, although incident before 1977 were interviewed sufficiently early in the study that their interval from presentation to interview was within that which could occur under the study protocol (i.e. 50 months, from January 1977 to February 1981). These patients – 2 cases, 5 radiotherapy controls and 1 non-radiotherapy control – have been included in the analyses presented here. A further 3 cases have been included in the analyses who were incident during the study period and treated at study centres, but were resident in areas just outside the study area (the catchments for the treatment centres did not have completely clear-cut borders in reality). The analyses therefore included 259 cases with testicular cancer. The histologies of these tumours, as stated in the pathology reports in the case notes, were: 138 seminoma, unmixed with other histological types; 104 teratoma, unmixed with other histological types; and 17 others, of mixed cell type or other cell types. This distribution reflects British classificatory practice for histology of testis cancer (Collins & Pugh, 1964) and hence several pathological types common in US studies were not so

Correspondence: A.J. Swerdlow at his present address: Office of Population Censuses and Surveys, St. Catherines House, 10 Kingsway, London WC2B 6JP.
Received 21 April 1986 and in revised form 6 August 1986.

classified in the present material. The age and histology distribution of the cases (Table I) was similar to that for the 469 testis cancers incident in the catchment population, except that the cases showed a deficit of elderly patients and of patients with tumours of unknown histology (none of the 5 individuals in the latter category were interviewed).

**Table I** Age at presentation of cases of testis cancer included in the case-control study, and of all patients with testis cancer resident in the study catchment area, January 1977–February 1981.

| *Age (years)* | *Cases* (%) | | | | *All testis cancers in catchment area* (%) |
|---|---|---|---|---|---|
| | *Teratoma* | *Seminoma* | *Other histologies* | *Total, all cases* | |
| <25 | 28 (27) | 6 (4) | 3 (18) | 37 (14) | 68 (14) |
| 25–34 | 48 (46) | 48 (35) | 5 (29) | 101 (39) | 169 (36) |
| 35–44 | 20 (19) | 52 (38) | 2 (12) | 74 (29) | 121 (26) |
| 45–54 | 4 (4) | 23 (17) | 1 (6) | 28 (11) | 57 (12) |
| 55–64 | 1 (1) | 5 (4) | 2 (12) | 8 (3) | 26 (6) |
| ≧65 | 3 (3) | 4 (3) | 4 (24) | 11 (4) | 28 (6) |
| Total, all ages | 104 (100) | 138 (100) | 17 (100) | 259 (100) | 469 (100) |

One control group was patients treated at the same radiotherapy centres as the cases, with any diagnosis except cancers of the testis, other genital organs, lung, and unknown primary site (the 'radiotherapy controls'). Two hundred and thirty-eight such controls were included in the analyses; all but 3 were patients with cancer, most commonly Hodgkin's disease (83 patients), non-Hodgkin's lymphoma (31), brain tumour (23) and bladder cancer (18). The other control group was in-patients from hospitals in the same towns as the radiotherapy centres, with non-malignant diseases excluding chronic diseases likely to affect lifestyle substantially (the 'non-radiotherapy controls'). Two hundred and fifty-one non-radiotherapy controls were included, with a wide range of general surgical, orthopaedic, dental and ENT conditions, most commonly deflected nasal septum (19 patients), disorders of tooth eruption (18), haemorrhoids (16), acute tonsillitis (15), varicose veins of lower extremities (13), nasal polyps (13), and appendicitis (13). An attempt was made to select controls such that, within each centre their age distribution was similar to that of the cases.

Cases and controls were interviewed in a similar manner using a structured interview schedule between April 1979 and March 1981 in hospitals (except in a very few instances where, for practical reasons, interviews were conducted at the patient's home or workplace); additional data were extracted from hospital case-notes. The interviewers were, where possible, 'blind' to the diagnoses of the cases and radiotherapy controls; such blindness was not possible for the non-radiotherapy controls. The subjects were not informed of the specific disease of interest in the study, and the questionnaire was constructed to give no obvious indication of the disease under investigation (for instance, enquiry about cryptorchidism was made in a question asking also about several other named congenital malformations). The non-radiotherapy controls were interviewed, on average, sooner after hospital presentation (4.4 months) than were the cases (12.6 months) or the radiotherapy controls (9.8 months). This occurred partly because the radiotherapy patients, unlike the non-radiotherapy controls, were often interviewed at out-patient follow-up, and partly because the radiotherapy patients usually presented to a surgeon but could only be interviewed when they were subsequently transferred to radiotherapy care. In order to compensate for this difference when calculating age at 'presentation', a pseudo-date of presentation for use in determining age was calculated for each non-radiotherapy control by subtracting from the patient's date of interview the mean duration from hospital presentation to interview for the cases treated at the same town.

The study investigated a large number of possible risk factors; this report concerns the disease, operation, and drug treatment associations of testicular cancer. Patients were asked about any serious or chronic illnesses, and any hospital admissions and operations, as well as specific questions about several named infections, complications of these infections, venereal diseases in the subject and his partner(s), several named congenital abnormalities, inguinal hernia, and (where appropriate) treatment of cryptorchidism. Questions were also asked about all drugs taken at least once per week for three months or more, about any medication with androgens, oestrogens and other hormones, and about taking of LSD. Information about illnesses and operations was also extracted from available case notes – usually in practice for testis cancer patients and radiotherapy controls the radiotherapy department notes rather than the entire hospital notes. The case notes rarely mentioned conditions not also mentioned in the interview; such mentions did not appear to be biased and were too few to have affected the results substantially. Case note information did sometimes allow clearer classification of conditions referred to in lay terms by the subject at interview. Case note and interview information have therefore been combined in the analyses presented.

Analysis was by conditional logistic regression using the computer program PECAN (Storer *et al.*, 1983), estimating relative risks after stratifying for age (using 2-year age groupings between 20 and 49 years and the additional groups <20, 50–54, 55–59, and ≧60 years) and two regions of residence (West Midlands region, and Oxford region including Cheltenham). This grouping of radiotherapy centre catchments was on the basis of similarity of demographic characteristics. The study data were examined with respect to risks of testicular cancer overall and of sub-divisions of testicular cancer by histology; the latter analyses have in general only been presented where they indicated risks for specific histologies different from those for testicular cancer overall. Risks were examined separately in comparison with each of the two control groups; such risks are presented in the tables. Where risks were found to be similar using each set of controls, the overall risks, based upon the two control groups combined, are the ones presented in the text. Controls who from their admission diagnoses were known to be biased for particular analyses were excluded from those analyses: thus, for example, a non-radiotherapy control who was interviewed during an admission for resuture of vas was excluded from analyses of risk associated with past vasectomy.

### Results

### *Cryptorchidism*

The main known risk factor from previous studies, cryptorchidism, gave a relative risk (RR) for testicular cancer overall of 6.3 (95% confidence limits (CL) = 2.9–13.9; seminoma RR = 6.8; teratoma RR = 5.7) (Table II). Fifteen of the 27 cases and 5 of the 9 controls with a history of cryptorchidism had undergone operation for maldescent, although in 2 cases and 2 controls the operation was known to have been unsuccessful (in one cases leading to subsequent orchidectomy). In a further 3 cases and 1 control spontaneous descent was stated to have occurred.

Only 2 cases had undergone successful orchidopexy before the age of 10 years. There were too few controls who had undergone the operation to analyse risk of malignancy by age at orchidopexy, but there was some indication that long uncorrected maldescent may be associated particularly with seminoma: 9 of the 11 cases with no history of spontaneous

**Table II** Relative risk of testicular cancer in relation to selected conditions.

| *Condition* | *Number (%) of of cases with risk factor* | *Number (%) of radiotherapy controls with risk factor* | *Number (%) of non-radiotherapy controls with risk factor* | *Relative risk, cases compared to radiotherapy controls* | *Relative risk, cases compared to non-radiotherapy controls* | *Relative risk (95% CL), cases compared to both control groups combined* |
|---|---|---|---|---|---|---|
| Cryptorchidism | 27 (10) | 6 (3) | 3 (1) | 4.7[c] | 10.5[c] | 6.3 (2.9–13.9)[c] |
| Inguinal hernia | 23 (9) | 18 (8) | 12 (5) | 1.5 | 1.9 | 1.6 (0.9–2.9) |
| hernia without cryptorchidism | 16 (6) | 16 (7) | 11 (4) | 1.2 | 1.7 | 1.3 (0.7–2.7) |
| herniorrhaphy before age 15 years | 8 (3) | 4 (2) | 3 (1) | 2.2 | 2.4 | 2.3 (0.8–6.7) |
| herniorrhaphy before age 15 years but no cryptorchidism | 4 (2) | 4 (2) | 3 (1) | 1.5 | 1.2 | 1.3 (0.4–4.7) |
| Mumps orchitis | 5 (2) | 1 (0) | 0 (0) | 6.0 | (∞) | 12.7 (1.4–113.6)[b] |
| Atopy | 30 (12) | 16 (7) | 17 (7)[d] | 1.6 | 2.1[a] | 1.8 (1.1–3.1)[a] |
| hay fever | 18 (7) | 5 (2) | 8 (3)[d] | 2.9[a] | 2.7[a] | 2.6 (1.2–5.6)[a] |
| eczema | 8 (3) | 4 (2) | 1 (0)[d] | 1.9 | 7.7[a] | 3.1 (1.0–10.0) |
| asthma | 14 (5) | 7 (3) | 9 (4)[d] | 1.7 | 1.7 | 1.7 (0.8–3.6) |
| Meningitis | 4 (2) | 1 (0) | 1 (0) | 2.6 | 3.9 | 3.0 (0.5–17.5) |
| Tuberculosis | 4 (2) | 2 (1) | 2 (1) | 3.3 | 2.2 | 2.7 (0.6–12.4) |
| Pneumonia | 16 (6) | 8 (3) | 14 (6) | 2.1 | 0.9 | 1.4 (0.7–2.8) |
| Total | 259 (100) | 238 (100) | 251 (100) | | | |

[a]$P<0.05$; [b]$P<0.01$; [c]$P<0.001$; [d]from 234 controls (17 eliminated because diagnosis related to risk factor).

or therapeutic descent of the cryptorchid testis (all aged over 20 years at presentation) were seminomas, as was the only case with orchidopexy after age 20 years.

Among the 18 cases with unilateral testis cancer and unilateral cryptorchidism of known side, in 11 the cancer was ipselateral to the maldescent and in 7 it was contralateral. The mean age at incidence of testis cancer was similar in cryptorchid men to that in non-cryptorchid men.

### *Inguinal hernia*

Inguinal hernia was associated with a relative risk for testicular cancer of 1.6 (NS) (Table II). Age at incidence of the cancer was not affected by hernia history. All but 2 (both seminomas) of the 23 cases and all but 1 of the 30 controls who had had herniae had undergone herniorrhaphy. Risk of testis cancer was raised for herniorrhaphy before age 15 years (RR = 2.3; NS) but not later (RR = 1.1); childhood herniorrhaphy was particularly associated with seminoma (RR = 3.8; $P<0.05$), but this was not the case for later herniorrhaphy. The age at first presentation of the hernia was rarely known.

Of the 18 cases with unilateral testicular cancer and ever-hernia of known side, the tumour occurred on the side of the hernia in 7, and on the opposite side in 11. Testicular cancers in men with herniae were predominantly left-sided (16 left-sided tumours, 6 right-sided and 1 of unknown side); this was due to a significant preponderance of left-sided seminomas (13 left-sided, 3 right-sided; $P<0.05$). No such preponderance was present in cases without herniae; 119 testis cancers were left-sided, 115 right-sided, and 2 bilateral.

Restricing the hernia analyses to men who never cryptorchid reduced the risks of testicular cancer for men with hernia (RR = 1.3; NS) and with herniorrhaphy before age 15 years (RR = 1.3; NS) but left a preponderance of left-side tumours (13 left-sided, 3 right-sided ($P<0.05$)).

### *Mumps orchitis*

Five cases (2 with seminoma, 3 with other histologies) and 1 control had histories of mumps orchitis (RR = 12.7; $P<0.01$). Mumps orchitis had occurred at age 15 years in one case, and over 20 years in the remainder. The side of orchitis was generally unknown, and only one case was known to have had consequent testicular atrophy.

A history of mumps was associated with a significantly raised risk when cases were compared to radiotherapy controls (RR = 1.59; 95% CL 1.04–2.45) but not when compared to non-radiotherapy controls (RR = 1.07; 95% CL 0.69–1.67) and not overall. Histories of other specific infections – chickenpox, glandular fever, measles, rubella and whooping cough – showed no significant association with testicular cancer, although for measles the risk was of borderline significance when comparison was with the radiotherapy controls (RR = 1.79; 95% CL 0.98–3.26) but not raised when comparison was with the non-radiotherapy controls (RR = 1.07; 95% CL 0.57–2.00). Mumps had occurred at age 15 years or over more often in cases (14 (7%)) than in radiotherapy controls (5 (3%)) or non-radiotherapy controls (8 (4%)); the excess partly reflected cases with adult mumps orchitis, noted above.

### *Other genital conditions*

Several other genital conditions showed non-significant excesses in cases. Hydrocoele was recorded for 6 cases (5 ipselateral to the tumour, 1 bilateral hydrocoeles) and 3 controls. Two of the cases and 2 of the controls had undergone operation for hydrocoele: the unoperated coeles in cases had been diagnosed at the same time as the cancer (1) or 1 or 2 years previously (2) or at an unknown date (1). Testicular atrophy without a history of mumps orchitis was recorded in 9 non-cryptorchid cases (3 with atrophy ipselateral, 6 contralateral to the tumour) and no controls, and epidydymitis or orchitis not known to be due to mumps in 4 further cases (all seminomas; all ipselateral to the tumour) but no controls. Hypospadias had been present in 1 case and 2 controls, and varicocoele in 2 cases (both with teratoma) and 3 controls. Testicular injury was not specifically enquired about, but was volunteered at interview by 6 cases and 1 control; none of these subjects had been admitted to hospital for the injury.

### *Atopy*

Atopy – taken as a history of asthma, hay fever or eczema – was associated with a significantly raised risk of testicular cancer (Table II: RR = 1.8; $P<0.05$). Risk was raised for each of asthma, hay fever and eczema separately (Table II), and was similarly raised for seminoma and teratoma. All but one of the asthmatic cases and all but 3 of the asthmatic controls were known to have had onset of asthma before age 15 years. Risk of testicular cancer was also raised for users for at least 3 months of some categories of drugs which can be used to treat atopy – steriods (RR = 1.9; NS), anti-histamines (RR = 1.2; NS), and adrenoceptor stimulants (RR = 2.0; NS); three cases but no controls stated that they had received a course of desensitising injections, and 2 cases and 4 controls had used disodium cromoglycate. There was no indication of raised risk for men using any of the above drugs for non-atopic indications.

For a few subjects, there was a record of long term treatment with a drug which can be used to treat atopic conditions, but no record of an atopic condition or other condition appropriate to the treatment. It seems likely that some of these subjects had had atopic conditions which they did not think merited mention when they were asked about diseases (there were no interview questions specifically about atopic conditions). The study records of such patients were therefore examined by a medical colleague unaware of the case-control status of the subjects; this identified 3 further cases and 1 further control who were probably atopic and an additional 2 cases and 1 control who probably had bronchitis with an asthmatic component (none of whom have been included in the above relative risk calculations).

*Other diseases*

There was an excess of cases with a history of meningitis (RR = 3.0; NS). The causative organism of meningitis was always unknown; the age at infection varied considerably. Non-significant excesses were also noted for histories of tuberculosis (RR = 2.7) and pneumonia (RR = 1.4), particularly pneumonia before age 5 years (RR = 1.7; NS), but not of bronchitis or other lung diseases. A history of epilepsy was recorded for 3 cases and 1 control; all of the cases had taken phenobarbitone, one had taken phenytoin, and one had taken sodium valproate. The 469 potential cases for the study included 2 patients with Down's syndrome and 8 others who were known to be mentally retarded.

Excepting the malformations discussed above, congenital abnormalities of the urinary tract were known in 1 case (a congenitally abnormal pelvic kidney found only during investigation of the testicular cancer) and 2 controls, and other congenital abnormalities (often minor or ill-specified, but including 2 cases with serious congenital eye abnormalities) in 10 cases and 10 controls. There was no raised risk for other diseases or abnormal symptons of the urinary tract, or for venereal diseases in the subjects or their partners. One case had had a previous malignancy (a bladder cancer), and a further 4 cases had had papillomata excised.

*Operations*

Risk in relation to some common operations is shown in Table III. The tumour was not strongly associated with any of these operations or the age at which they were performed. Risks in relation to orchidopexy, herniorraphy and hydrocoele operations have been discussed above; no other operation was associated with risk of the tumour.

*Drugs*

There was no clear increased risk of testicular cancer associated with chronic use of any drug not discussed above, nor with ever-use of androgens (1 case, 3 controls), oestrogens (possibly 1 case, 1 control) or LSD (8 cases, 2 radiotherapy controls, 8 non-radiotherapy controls).

### Discussion

Cryptorchidism was found to be a major risk factor for testicular cancer in this study, with a relative risk (6.3) which is consistent with most previous epidemiologic findings. The present results suggest that any greater risk of seminoma than of teratoma in cryptorchidism may be due to a particularly raised risk of seminoma associated with prolonged uncorrected maldescent. It is consistent with this that in past studies which showed greater risk of seminoma than of teratoma in cryptorchid patients (Collins & Pugh, 1964; Miller & Seljelid, 1971; Morrison, 1976) the majority of the cryptorchid subjects had had no successful treatment. Also, data from a large clinical series (Batata *et al.*, 1982) were in the direction of particular risk of seminoma in uncorrected cryptorchidism.

Testicular cancer in unilaterally cryptorchid males occurred more often in the maldescended than in the normally descended testis. Adding together the present and previous published laterality data for men with unilateral testicular cancer and unilateral cryptorchidism (Thurzó & Pintér 1961; Field, 1962; Collins & Pugh, 1964; Johnson *et al.*, 1968; Gehring *et al.*, 1974; Morrison, 1976; Henderson *et al.*, 1979; Schottenfeld *et al.*, 1980; Herman *et al.*, 1981; Batata *et al.*, 1982; Coldman *et al.*, 1982; Pottern *et al.*, 1985) gives a total of 318 tumours ipselateral and 65 tumours contralateral to maldescent – i.e. a risk 4.9 times higher in the undescended than in the descended testis. This implies that in comparison to the risk of malignancy in descended testes generally, the risk in a descended testis opposite maldescent is about $1\frac{1}{2}$ to 3 times raised (depending upon the estimate used for overall risk of testis cancer in a cryptorchid man). Totalling the present histology-specific data and previous results published in sufficient detail (Thurzó & Pintér, 1961; Field, 1962; Collins & Pugh, 1964; Johnson *et al.*, 1968; Gehring *et al.*, 1974; Herman *et al.*, 1981; Coldman *et al.*, 1982), suggests that the risk of malignancy in a cryptorchid testis compared to that in a contralateral descended testis is similar for seminoma ($n = 80$, ratio of risks = 4.3) and for non-seminoma histologies ($n = 136$, ratio = 3.9).

**Table III** Relative risk of testicular cancer in relation to selected operations.

| *Operation* | *Number (%) of cases with risk factor* | *Number (%) of radiotherapy controls with risk factor* | *Number (%) of non-radiotherapy controls with risk factor*[a] | *Relative risk (95% CL), cases compared to radiotherapy controls* | *Relative risk (95% CL), cases compared to non-radiotherapy controls* | *Relative risk (95% CL), cases compared to both control groups combined* |
|---|---|---|---|---|---|---|
| Tonsils and adenoids operations | 73 (28) | 60 (25) | 63 (27) | 1.10 (0.72–1.67) | 1.10 (0.72–1.68) | 1.07 (0.75–1.52) |
| Circumcision and preputiotomy | 47 (18) | 54 (23) | 54 (22) | 0.69 (0.43–1.10) | 0.67 (0.41–1.08) | 0.69 (0.46–1.03) |
| Ligation of vas or vasectomy | 22 (8) | 16 (7) | 20 (8) | 1.28 (0.62–2.63) | 0.99 (0.50–1.98) | 1.13 (0.63–2.04) |
| Operations on appendix | 31 (12) | 29 (12) | 34 (14) | 0.92 (0.52–1.63) | 0.78 (0.45–1.36) | 0.88 (0.55–1.42) |
| Total | 259 (100) | 238 (100) | 251 (100) | | | |

[a]excluding for each operation, controls whose current admission was for the same or a related operation (i.e. 17 tonsils and adenoids, 2 circumcision, 1 resuture of vas, 12 operations on appendix).

Full consideration of risk of testis cancer in cryptorchidism needs also to take account of the position of maldescent and the nature and age of any treatment of the condition; our study had insufficient numbers for any confident conclusion on risks related to these issues. The above risk estimates using varied sources of data are necessarily crude and open to potential bias; furthermore, case-control study and clinical series data on risk of malignancy in cryptorchidism are problematic because of the difficulty of determining reliably the past position of testes which are not maldescended at the time of adult presentation. Subjects may not know of cryptorchidism if spontaneous descent or successful treatment occurred at a young age, or they may believe that they have been cryptorchid when in fact they have had retracted testes or misdiagnosed normally descended testes. A cohort study of malignancy in cryptorchidism could provide more reliable data.

The relative risk of adult testis cancer found for men who had ever had an inguinal hernia (1.6) was compatible with that from previous studies (Henderson *et al.*, 1979; Schottenfeld *et al.*, 1980; Mills *et al.*, 1984; Pottern *et al.*, 1985). There is evidence that inguinal hernia is associated with childhood testis cancer also (Li & Fraumeni, 1972; Swerdlow *et al.*, 1982). The present study, and a study confined to seminomas (Coldman *et al.*, 1982), suggested a higher risk of adult testis cancer for men with a history of childhood hernia than for those whose herniae manifested in adulthood. This accords with the slightly higher risk of testis cancer found in studies restricted to childhood herniae (Morrison, 1976; Depue *et al.*, 1983) than in studies including herniae at any age (Henderson *et al.*, 1979; Schottenfeld *et al.*, 1980; Mills *et al.*, 1984). Pottern *et al.* (1985), however, found greatest risk for men with greatest age at herniorrhaphy, although this was in a study where most of the herniorrhaphies were in childhood.

The present work suggested that childhood hernia may particularly be a risk factor for seminoma, as did the results of Coldman *et al.* (1982) but not Morrison (1976).

As in some (Morrison, 1976; Henderson *et al.*, 1979) but not all (Gehring *et al.*, 1974; Pottern *et al.*, 1985) previous studies, the side of testicular cancer was not related to the side of inguinal hernia/herniorrhaphy in the present material.

Inguinal hernia is associated with undescended testis (Scorer & Farrington, 1971; Marshall, 1982) and on this basis alone some association between hernia and testis cancer would be expected. The extent to which this explains the raised risk of testis cancer associated with hernia is unclear: in analyses of risk associated with hernia excluding subjects with known cryptorchidism, Pottern *et al.* (1985) and the present study found relative risks of 1.3, but relative risks of about 3 were found in a study restricted to seminomas (Coldman *et al.*, 1982) and a study restricted to herniae repaired in childhood (Morrison, 1976).

Mumps orchitis appears to be a risk factor for testis cancer, but not a major one. Kaufman & Bruce (1963) identified reports of 28 cases of testicular cancer following mumps orchitis. Beard *et al.* (1977*a*) found 2 testicular cancers in follow-up of 132 men with a history of mumps orchitis, while in case-control studies Lin and Kessler (1979) and the present study found significant risks of testis cancer for men with mumps orchitis, and Mills *et al.* (1984) found a significant risk for men with a history of orchitis (cause(s) not specified in the publication). The balance of evidence does not strongly suggest, however, that mumps without clinical orchitis raises risk of the tumour: Morrison (1976) and Henderson *et al.* (1979) found no increase in risk for men with a history of mumps, there was not a clear increase in the present study, whilst Loughlin *et al.* (1980) found a borderline significant raised risk for such men. Mumps orchitis can cause permanent damage to the germinal epithelium and to Leydig-cell function (Adamopoulos *et al.*, 1978), and is more likely to cause tubular damage when it occurs in adults rather than before puberty (Schottenfeld & Warshauer, 1982). It is notable that all of the cases with a history of mumps orchitis in the present study had had the infection after childhood.

Testicular atrophy also appears to be associated with testicular cancer. Microscopic and clinical atrophy occur frequently in cryptorchidism (Scorer & Farrington, 1971). Cancer *in situ* of the testis occurs in testes which are on average smaller than normal, and, in unilateral cancer *in situ*, smaller than the contralateral testis (Skakkebaek *et al.*, 1982). In the present study all 9 subjects with atrophy but not cryptorchidism or mumps orchitis were cases, although there was clearly potential for reporting bias. In previous series without control data, up to 40% of testis cancers have occurred in atrophic testes (Hausfeld & Schrandt, 1965; Beard *et al.*, 1977*b*; Ehrengut & Schwartau, 1977) and 6% in testes opposite atrophy (Ehrengut & Schwartau, 1977).

The present study is the third to show a raised risk of testis cancer for men with hydrocoele (Schottenfeld *et al.*, 1980; Mills *et al.*, 1984). In neither of the previous reports was it clear how frequently the diagnosis of hydrocoele had preceded the testicular cancer, and in the present study there were very few cases with hydrocoele clearly preceding the tumour; the association may be due to bias.

Congenital genito-urinary defects other than those above appear to be associated with testicular cancer in children (Li & Fraumeni, 1972; Sakashita *et al.*, 1980; Swerdlow *et al.*, 1982), but there was no evidence for such an association in adults in the present study, and only one previous study has given evidence which might suggest raised risk in adults (Henderson *et al.*, 1979).

An unexpected result was the significant association of testicular cancer with a history of atopy. The analysis of atopy was restricted to asthma, hay fever and eczema because it was not possible from the available histories to infer the atopic status of individuals who had reported other conditions such as drug 'allergies' and unspecified urticaria which might have an atopic basis in some instances. There were comparatively few individuals with these other conditions, however, and their inclusion would not substantially have altered the results. Asthma can occur without an atopic basis in association with chronic bronchitis, generally at older ages, but in the present findings onset of asthma had generally been in childhood and none of the asthmatics gave a history of bronchitis or other chronic obstructive airways disease. The information on atopic conditions was taken from the interview questions and case note data about serious and chronic illnesses in general. Atopy may well have been under-recorded in both cases and controls, since the study did not specifically enquire about any atopic conditions by name (there was no prior hypothesis of an association) and some subjects might well have regarded atopic conditions as too trivial to volunteer at interview; since the interview asked about all chronic drug treatments, however, few if any patients with severe atopy should have been missed. Biased under-reporting or under-recording of atopic conditions is improbable since the hypothesis of an association appears not to have been published previously and is unlikely to have been suspected by the subjects or interviewers; the cases did not have a substantial tendency to disclose more diseases in general than did the controls; similar raised risks for atopy were found for each set of controls; and raised risks were found for each atopic condition analysed separately. No potential condounding variables for the association were apparent; no one type of drug treatment of atopy was associated with a particularly high risk.

Previous data on atopy and testis cancer are limited. A cohort study of male asthmatics (Robinette & Fraumeni, 1978) found three testis cancer deaths compared to two expected. In case–control studies, each with many fewer cases than the present study, but each probably with confidence limits compatible with the present findings, Henderson *et al.* (1979) found no raised risk of testicular cancer for asthma or for hay fever, whilst Vena *et al.* (1985) found non-significant relative risks of about 1.6 for testis cancer in relation to histories of each of asthma, hay fever, and hives – the highest risks they found for any cancer site in males.

The risks of testicular cancer in men with meningitis does not appear to have been investigated previously. Maternal tuberculosis has previously been found a significant risk factor for childhood testicular cancer (Swerdlow *et al.*, 1982). A history of pneumonia, which was a slight risk factor in the present study, was significantly associated with risk of testis cancer in a US cohort study (Whittemore *et al.*, 1985). Like the association with atopy, there is no reason to believe that bias or confounding explained the associations with meningitis, tuberculosis and pneumonia. The available evidence from the literature, discussed above, gives some support to the notion that the associations found with atopy, tuberculosis and pneumonia may not merely be chance findings, although clearly further investigation in other data sets is required. A possible aetiological pathway can be suggested which would link testis cancer to atopy, meningitis, tuberculosis and pneumonia. Patients with atopy probably have deficiencies in T-cell immunity (Buckley & Becker, 1978; Strannegård & Strannegård, 1978). T-cells are also important in defence against tuberculosis (Chaparas, 1982) and T-cell deficiency may be associated with an increased susceptibility to tuberculosis – in several conditions which include suppressed cell mediated immunity there is an excess of tuberculosis (Chaparas, 1982). There is also evidence that cell mediated immunity, including T-cells, is of importance in immunity to viruses causing respiratory infections (Ganguly & Waldman, 1982) and meningitis (Welliver *et al.*, 1982). The T-cell is though to play a central role in the immune response to neoplasia (Rosenbaum & Dwyer, 1977), and epidemiological evidence suggests (Kinlen, 1982) that immunological factors are important in the development of some but not most cancers in man. Testis cancer has in common with several of these tumours that it does not increase steeply in incidence with age. Cases of testis cancer have been reported in men immunosuppressed after renal transplantation (Nellans & Ravera, 1975; Cabrera *et al.*, 1976) and in homosexual men with cellular immune deficiency (Logothetis *et al.*, 1985), but excesses of testis cancer in follow-up of such men have not at present been reported.

The evidence that atopy and certain infections are associated with testicular cancer incidence needs replication. Also, it may be worthwhile to investigate in aetiological studies the immunological status of patients with early testicular cancer.

We thank the Cancer Research Campaign for funding the study; Professor Sir Richard Doll, Professor M.P. Vessey and Dr P. Amlot for advice, and Professor Vessey and Dr E.R. Rue for support; the clinicians, their staff and others, too numerous to thank individually here, who cooperated and helped with the study and provided notification of cases; Mrs M.A. Ainley and Miss M.M. Stone for their sensitive and skilful interviewing; Mrs F. Garven, Miss J. Clark, and Mr A. Radalowicz for computer programming; and Ms L. Semke for secretarial assistance.

### References

ADAMOPOULOS, D.A., LAWRENCE, D.M., VASSILOPOULOS, P., CONTOYIANNIS, P.A. & SWYER, G.I.M. (1978). Pituitary–testicular interrelationships in mumps orchitis and other viral infections. *Br. Med. J.*, **1**, 1177.

BATATA, M.A., CHU, F.C.H., HILARIS, B.S., WHITMORE, W.F. & GOLBEY, R.B. (1982). Testicular cancer in cryptorchids. *Cancer*, **49**, 1023.

BEARD, C.M., BENSON, R.C. JR., KELALIS, P.P., ELVEBACK, L.R. & KURLAND, L.T. (1977a). The incidence and outcome of mumps orchitis in Rochester, Minnesota, 1935 to 1974. *Mayo Clin. Proc.*, **52**, 3.

BEARD, C.M., BENSON, R.C. JR., KELALIS, P.P. & ELVEBACK, L.R. (1977b). Incidence of malignant testicular tumors in the population of Rochester, Minnesota, 1935 through 1974. *Mayo Clin. Proc.*, **52**, 8.

BUCKLEY, R.H. & BECKER, W.G. (1978). Abnormalities in the regulation of human IgE synthesis. *Immunological Rev.*, **41**, 288.

CABRERA, R.C., BOHORQUEZ, J.F., KINKHABURALA, R. & KOUNTZ, S.L. (1976). Mixed testicular tumor in immunosuppressed patient: case report. *J. Urol.*, **116**, 823.

CHAPARAS, S.D. (1982). Immunity in tuberculosis. *Bull. World Health Organization*, **60**, 447.

COLDMAN, A.J., ELWOOD, J.M. & GALLAGHER, R.P. (1982). Sports activities and risk of testicular cancer. *Br. J. Cancer*, **46**, 749.

COLLINS, D.H. & PUGH, R.C.B. (1964). Classification and frequency of testicular tumours. *Br. J. Urol.*, Suppl. to **36**, 1.

DEPUE, R.H., PIKE, M.C. & HENDERSON, B.E. (1983). Estrogen exposure during gestation and risk of testicular cancer. *J. Natl Cancer Inst.*, **71**, 1151.

EHRENGUT, W. & SCHWARTAU, M. (1977). Mumps orchitis and testicular tumours. *Br. Med. J.*, **ii**, 191.

FIELD, T.E. (1962). Malignancy in the ectopic testicle in army patients. *J. Royal Army Med. Corps*, **108**, 189.

GANGULY, R. & WALDMAN, R.H. (1982). Immunology of respiratory viruses. In: *Immunology of Human Infection, Part II: Viruses and Parasites; Immunodiagnosis and Prevention of Infectious Diseases*, Nahmias & O'Reilly (eds), p. 165. Plenum: New York.

GEHRING, G.G., RODRIGUEZ, F.R. & WOODHEAD, D.M. (1974). Malignant degeneration of cryptorchid testes following orchiopexy. *J. Urol.*, **112**, 354.

HAUSFELD, K.F. & SCHRANDT, D. (1965). Malignancy of testis following atrophy: Report of three cases. *J. Urol.*, **94**, 69.

HENDERSON, B.E., BENTON, B., JING, J., YU, M.C. & PIKE, M.C. (1979). Risk factors for cancer of the testis in young men. *Int. J. Cancer*, **23**, 598.

HERMAN, J.G., HAWKINS, N.V., RIDER, W.D. & CROSS CANADA TESTIS AUDIT GROUP. (1981). Cryptorchidism and non-seminomatous testis cancer. *Int. J. Androl.*, Suppl. **4**, 123.

JOHNSON, D.E., WOODHEAD, D.M., POHL, D.R. & ROBISON, J.R. (1968). Cryptorchism and testicular tumorigenesis. *Surgery*, **63**, 919.

KAUFMAN, J.J. & BRUCE, P.T. (1963). Testicular atrophy following mumps. A cause of testis tumour? *Br. J. Urol.*, **35**, 67.

KINLEN, L.J. (1982). Immunologic factors. In: *Cancer Epidemiology and Prevention*, Schottenfeld and Fraumeni (eds), p. 494. W.B. Saunders: Philadelphia.

LI, F.P. & FRAUMENI, J.F. JR. (1972). Testicular cancers in children: Epidemiologic characteristics. *J. Natl Cancer Inst.*, **48**, 1575.

LIN, R.S. & KESSLER, I.I. (1979). Epidemiologic findings in testicular cancer. *Am. J. Epidemiol.*, **110**, 357.

LOGOTHETIS, C.J., NEWELL, G.R. & SAMUELS, M.L. (1985). Testicular cancer in homosexual men with cellular immune deficiency: Report of 2 cases. *J. Urol.*, **133**, 484.

LOUGHLIN, J.E., ROBBOY, S.J. & MORRISON, A.S. (1980). Risk factors for cancer of the testis. *N. Engl. J. Med.*, **303**, 112.

MARSHALL, F.F. (1982). Anomalies associated with cryptorchidism. *Urol. Clin. N. America*, **9**, 339.

MILLER, A. & SELJELID, R. (1971). Histopathologic classification and natural history of malignant testis tumors in Norway, 1959–1963. *Cancer*, **28**, 1054.

MILLS, P.K., NEWELL, G.R., JOHNSON, D.E. (1984). Testicular cancer associated with employment in agriculture and oil and natural gas extraction. *Lancet*, **i**, 207.

MORRISON, A.S. (1976). Cryptorchidism, hernia, and cancer of the testis. *J. Natl Cancer Inst.*, **56**, 731.

NELLANS, R.E. & RAVERA, J. (1975). Seminoma in a renal transplant recipient. *J. Urol.*, **113**, 871.

POTTERN, L.M., BROWN, L.M., HOOVER, R.N. *et al.* (1985). Testicular cancer risk among young men: role of cryptorchidism and inguinal hernia. *J. Natl Cancer Inst.*, **74**, 377.

ROBINETTE, C.D. & FRAUMENI, J.F. JR (1978). Asthma and subsequent mortality in World War II veterans. *J. Chron. Dis.*, **31**, 619.

ROSENBAUM, J.T. & DWYER, J.M. (1977). The role of IgE in the immune response to neoplasia: A review. *Cancer*, **39**, 11.

SAKASHITA, S., KOYANAGI, T., TSUJI, I., ARIKADO, K. & MATSUNO, T. (1980). Congenital anomalies in children with testicular germ cell tumor. *J. Urol.*, **124**, 889.

SCORER, C.G. & FARRINGTON, G.H. (1971). *Congenital Deformities of the Testis and Epididymis*. Butterworths: London.

SCHOTTENFELD, D. & WARSHAUER, M.E. (1982). Testis. In *Cancer Epidemiology and Prevention*, Schottenfeld & Fraumeni (eds), p. 947. W.B. Saunders: Philadelphia.

SCHOTTENFELD, D., WARSHAUER, M.E., SHERLOCK, S., ZAUBER, A.G., LEDER, M. & PAYNE, R. (1980). The epidemiology of testicular cancer in young adults. *Am. J. Epidemiol.*, **112**, 232.

SKAKKEBAEK, N.E., BERTHELSEN, J.G. & MÜLLER, J. (1982). Carcinoma-*in-situ* of the undescended testis. *Urol. Clin. N. America*, **9**, 377.

STORER, B.E., WACHOLDER, S. & BRESLOW, N.E. (1983). Maximum likelihood fitting of genral risk models to stratified data. *Appl. Statistics*, **32**, 172.

STRANNEGÅRD, O. & STRANNEGÅRD, I.-L. (1978). T-lymphocyte numbers and function in human IgE-mediated allergy. *Immunol. Rev.*, **41**, 149.

SWERDLOW, A.J., STILLER, C.A. & KINNIER WILSON, L.M. (1982). Prenatal factors in the aetiology of testicular cancer: An epidemiologic study of childhood testicular cancer deaths in Great Britain, 1953–73. *J. Epidemiol. Community Health*, **36**, 96.

THURZÓ, R. & PINTÉR, J. (1961). Cryptorchism and malignancy in men and animals. *Urol. Int.*, **11**, 216.

VENA, J.E., BONA, J.R., BYERS, T.E., MIDDLETON, E. JR., SWANSON, M.K. & GRAHAM, S. (1985). Allergy-related diseases and cancer: An inverse association. *Am. J. Epidemiol.*, **122**, 66.

WELLIVER, R.C., DRUCKER, M.M. & OGRA, P.L. (1982). Immunology of enteroviruses. In *Immunology of Human Infection, Part II: Viruses and Parasites; Immunodiagnosis and Prevention of Infectious Diseases*, Nahmias & O'Reilly (eds), p. 185. Plenum: New York.

WHITTEMORE, A.S., PAFFENBARGER, R.S. JR., ANDERSON, K. & LEE, J.E. (1985). Early precursors of site-specific cancers in college men and women. *J. Natl Cancer Inst.*, **74**, 43.